# Publisher Correction: Increasing ambient temperature progressively disassembles *Arabidopsis* phytochrome B from individual photobodies with distinct thermostabilities

**DOI:** 10.1038/s41467-020-16008-y

**Published:** 2020-04-20

**Authors:** Joseph Hahm, Keunhwa Kim, Yongjian Qiu, Meng Chen

**Affiliations:** Department of Botany and Plant Sciences, Institute for Integrative Genome Biology, University of California, Riverside, CA 92521 USA

**Keywords:** Nuclear organization, Light responses, Plant cell biology

Correction to: *Nature Communications* 10.1038/s41467-020-15526-z, published online 2 January 2020.

The original version of this Article contained an error in the title, which was previously incorrectly given as ‘Increasing ambient temperature progressively disassemble Arabidopsis phytochrome B from individual photobodies with distinct thermostabilities’. The correct version states ‘disassembles’ in place of ‘disassemble’. This has been corrected in both the PDF and HTML versions of the Article.

